# Description of *Spinocephalus tessellatus* n. gen., n. sp. (Rhabditida, Cephalobidae) from Iran, a nematode with a new morphological pattern at lip region

**DOI:** 10.21307/jofnem-2021-078

**Published:** 2021-10-01

**Authors:** Joaquín Abolafia, Manouchehr Hosseinvand, Ali Eskandari

**Affiliations:** 1 Departamento de Biología Animal, Biología Vegetal y Ecología, Universidad de Jaén, Campus ‘Las Lagunillas’ s/n, Edificio B3, 23071 Jaén, Spain; 2 Department of Plant Protection, Faculty of Agriculture, University of Zanjan, 45371-38791, Zanjan, Iran

**Keywords:** 18S rDNA, 28S rDNA, *Acromoldavicus skrjabini*, Description, Molecular analysis, Morphology, New genus, New species, Phylogeny, SEM, Taxonomy

## Abstract

A new genus and new species of the family Cephalobidae, subfamily Cephalobinae, named *Spinocephalus tessellatus* n. gen., n. sp. is described from Iran. Body 0.55–0.67 mm long, cuticle with tessellations, lateral field with two tessellated longitudinal wings, lip region with six triangular lips, primary axils deep and V-shaped with two conoid-elongate guard processes that originate from each lip, secondary axils deep and U-shaped with one thorn-like process (labial probolae?) in lateral view with a small rounded protuberance fused to the oral plate having triradiate symmetry more developed at the acute margin toward each primary axil, oral opening hexagonal, amphids large and clearly rounded to slightly oval, stoma cephaloboid with cheilostom with minute and rounded rhabdia, pharynx cephaloboid with corpus subcylindrical and isthmus very long being 1.4–1.7 times corpus length, nerve ring surrounds the isthmus, excretory pore at the level of the isthmus. Female monodelphic-prodelphic, spermatheca as long as the body diam., post-vulval uterine sac 0.8–1.0 times body diameter, tail conoid with small rounded terminus. Male monorchic, spicules 24–26 µm long, gubernaculum 11–14 µm long, tail conical and ventrally curved with small rounded terminus. Morphological, including SEM observations, and molecular (based on 18S and 28S rDNA) analyses revealed its relationship with the genera *Acromoldavicus* and *Nothacrobeles*.

The superfamily Cephaloboidea Filipjev, 1934, characterized with a monodelphic-prodelphic reproductive system with sac-like spermatheca, includes six families, three of which are divided in two subfamilies (Andrássy, 2005; Shokoohi and Abolafia, 2019) Alirhabditidae Suryawanshi, 1971 with a long tubular stoma and lacking labial probolae; Bicirronematidae Andrássy, 1978 having stoma with well-developed cheilostom and with labial cirri; Cephalobidae Filipjev, 1934 with short cheilostom including Acrolobinae De Ley, Siddiqi and Boström, 1993 without labial probolae and Cephalobinae Filipjev, 1934 with three labial probolae of variable morphology; Daubayliidae Chitwood and Chitwood, 1934 including a parasite of snails with a reduced stoma; Elaphonematidae Heyns, 1962a with irregular lips and reduced stoma including Elaphonematinae Heyns, 1962a with bilaterally symmetric lip region and Kirjanoviinae Andrássy, 1976 with three nearly triangular labial probolae; Osstellidae Heyns, 1962b with fused lips and poorly developed basal bulb including Osstellinae Heyns, 1962b with very short stoma and Drilocephalobinae (Ali, Suryawanshi and Chisty, 1973) with a nearly absent stoma.

In the present study, a new genus and species are described from Iran that has morphological and molecular features that are in between the families Cephalobidae and Elaphonematidae. Additionally, a related species, *Acromoldavicus skrjabini* (Nesterov, 1970) Nesterov and Lisetskaya, 1965 from Spain (Elaphonematidae, Kirjanoviinae), is described from SEM studies to compare the morphology of the two species.

## Materials and methods

### Nematode extraction and processing

The nematodes were extracted with a modified Baermann tray method (Whitehead and Hemming, 1965), killed and fixed by hot FPG (4:1:1, formaldehyde: propionic acid: glycerol), processed to anhydrous glycerol (Grisse, 1969), and mounted on glass microscope slides.

### Light microscopy (LM)

Photomicrographs were taken with a Nikon Eclipse 80i (Nikon, Tokyo, Japan) microscope with a differential interference contrast (DIC) optics mounted with a Nikon Digital Sight DS-U1 camera and processed with Adobe^®^ Photoshop^®^ CS. Demanian indices (de Man, 1881) and other ratios were calculated. The terminology used to describe the morphology of stoma and spicules-gubernaculum follows De Ley et al. (1995) and Abolafia and Peña-Santiago (2017), respectively.

### Scanning electron microscopy (SEM)

Specimens preserved in glycerine were selected and prepared for observation with a SEM according to Abolafia (2015). They were cleaned in distilled water, dehydrated in a graded ethanol-acetone series, critical point dried, coated with gold, and observed with a Zeiss Merlin microscope (5 kV) (Zeiss, Oberkochen, Germany).

### DNA extraction, PCR, and sequencing

Nematode DNA was extracted from single individuals, previously fixed in 70% ethanol, using a modified DNA extraction and PCR assays described by Castillo et al. (2003) somewhat modified (Archidona-Yuste et al., 2016). The specimens were cut in small pieces using the acute tip of a sterilized dental anesthesia needle on a clean slide with 18 ml of TE buffer (10 mM Tris-Cl + 0.5 mM EDTA; pH 9.0), transferred to a microtube and adding 2 μl proteinase K (700 μg/ml^‒1^) (Roche, Basel, Switzerland), and stored to –80°C within 15 min (for several days) until processing. Finally, the microtubes were incubated at 65°C (1 hr), then at 95°C (15 min) and the solution were use as DNA template. For DNA amplification, 3 μl of the extracted DNA was transferred to a microtube containing: 0.6 μl of each primer (10 mM), 3 μl Master Mix Taq DNA Polymerase (5x Hot FirePol Blend Master Mix) and ddH2O to a final volume of 20 μl. The primers used for amplification of the region of 18S rRNA gene were the forward primer SSU F_04 (5′-GCTTGTCTCCAAAGATTAAGCC-3′) and the reverse primer SSU R_26 (5′-CATTCTTGGCAAATGCTTTCG-3′) (Blaxter et al., 1998). The primers used for amplification of the D2-D3 region of 28S rRNA gene were the D2A (5′-ACAAGTACCGTGAGGGAAAGTTG-3′) and the D3B (5′-TCGGAAGGAACCAGCTACTA-3′) primers (De Ley et al., 1999; Nunn, 1992). PCR cycle conditions were as follows: one denaturation cycle of 94°C for 15 min., followed by 35 cycles of 94°C for 45 sec; annealing cycle of 55°C for 45 sec; extension cycle of 72°C for 45 sec, and finally one extension cycle of 72°C for 5 min. After DNA amplification, 5 μl of product was loaded on a 1% agarose gel in 0.5% Tris-acetate-EDTA (40 mM Tris, 20 mM glacial acetic acid and 2 mM EDTA; pH = 8) to verify the amplification using an electrophoresis system (Labnet Gel XL Ultra V–2, Progen Scientific, London, UK). The bands were stained with RedSafe (20,000x) previously added to the agarose gel solution. The sequencing reactions of the PCR products were performed at Sistemas Genómicos (Paterna, Valencia, Spain) according the Sanger et al. (1977) method. The rDNA sequences obtained for *Spinocephalus tessellatus* n. gen., n. sp. were submitted to the GenBank database.

### Phylogenetic analyses

For phylogenetic relationships, analysis was based on 18S and 28S rDNA. The newly obtained sequences were manually edited using BioEdit 7.2.6 (Hall, 1999) and aligned with other 28S rRNA gene sequences available in GenBank using ClustalW (Thompson et al., 1994) alignment tool implemented in the MEGA7 (Kumar et al., 2016). Alignments ends were trimmed using MEGA7. The best-fit model of nucleotide substitution used for the phylogenetic analysis was statistically selected using jModelTest 2.1.10 (Darriba et al., 2012). A phylogenetic trees were generated with the Bayesian inference method using MrBayes 3.2.6 (Ronquist et al., 2012). *Drilocephalobus* sp. (AY284680) for the 18S tree and *Deficephalobus desenderi* De Ley and Coomans, 1990 (GU062820) for the 28S tree was chosen as the outgroup. The analysis under GTR + I + G model was initiated with a random starting tree and run with the Markov Chain Monte Carlo (MCMC) (Larget and Simon, 1999) for 1 × 10^6^ generations. The tree was visualized and saved with FigTree 1.4.4 (Rambaut, 2018).

## Results

### Systematics

*Spinocephalus* n. gen.

### Diagnosis

Cephalobidae, Cephalobinae. Cuticle with tessellations, lateral field with two tessellated wings. Lip region with six triangular lips bearing two conoid-elongate processes at primary axils and one thorn-like process (labial probolae?) at secondary axils bearing a small rounded protuberance fused at oral plate with triradiate symmetry, oral opening surrounded by a hexagonal margin, amphids large and clearly rounded to slightly oval, stoma cephaloboid with cheilostom bearing minute and rounded rhabdia. Pharynx cephaloboid with corpus subcylindrical and isthmus unusually very long. Nerve ring and excretory pore at isthmus level. Female monodelphic-prodelphic with spermatheca well developed and post-vulval uterine sac poorly developed. Female tail conoid with rounded terminus. Male monorchic with spicules paired and symmetrical and gubernaculum well-developed. Male tail conical and ventrally curved with rounded terminus.

### Relationships

The new genus *Spinocephalus* n. gen. resembles, morphologically, other genera of the superfamily Cephaloboidea that have cuticle divided in blocks and arranged into longitudinal crests such as *Acromoldavicus* Nesterov, 1970, *Penjatinema* Heyns and Swart, 1998, and *Stegelleta* Thorne, 1938.

According to observations of SEM studies (Baldwin et al., 2001; Karegar et al., 1997, 1998; Susulovsky et al., 2001), the new genus *Spinocephalus* is distinguished from *Acromoldavicus* by having lips divided into plates with one long, acute process at primary axils and one large thorn-like process curved toward the oral opening at secondary axils (vs expanded lips with acute tips at primary axils and rounded at secondary axils), oral opening surrounded by a triacute margin with tips directed toward the primary axils and bearing three small rounded protuberance, one dorsal and two subdorsal (vs surrounded by three triangular labial probolae). Likewise, the new genus is different from *Penjatinema* (Heyns and Swart, 1998; Holovachov et al., 2009) by the lip region morphology (vs lips with fimbriated margin and oral opening surrounded by three long labial probolae with dendriform distal part). Finally, *Spinocephalus* n. gen. can be differentiate from *Stegelleta* (SEM by Boström and Holovachov, 2012, 2014; Orselli and Vinciguerra, 2002) also by the lip region morphology (vs fused lips in pairs with smooth margin or having short acute tip at primary axils and oral opening surrounded by three bifurcate labial probolae with smooth prongs. Molecularly, only *Acromoldavicus* presents closer relationships with *Spinocephalus* n. gen.

On the other hand, the morphology of the lip region resembles *Chilodellus* Boström and Holovachov, 2012 with irregular lips and unusually large amphids. However, labial probolae are very different, having bifurcate distal halves with convergent prongs in *Chilodellus*. Unfortunately, molecular studies are not available to confirm this relationship.

### Etymology

The generic name refers to the presence of acute processes (Latin *spina* = thorn) on the lip region (Latin *cephalus* from Greek *kephale* = head).

### Type and only species

**Figure 1:**
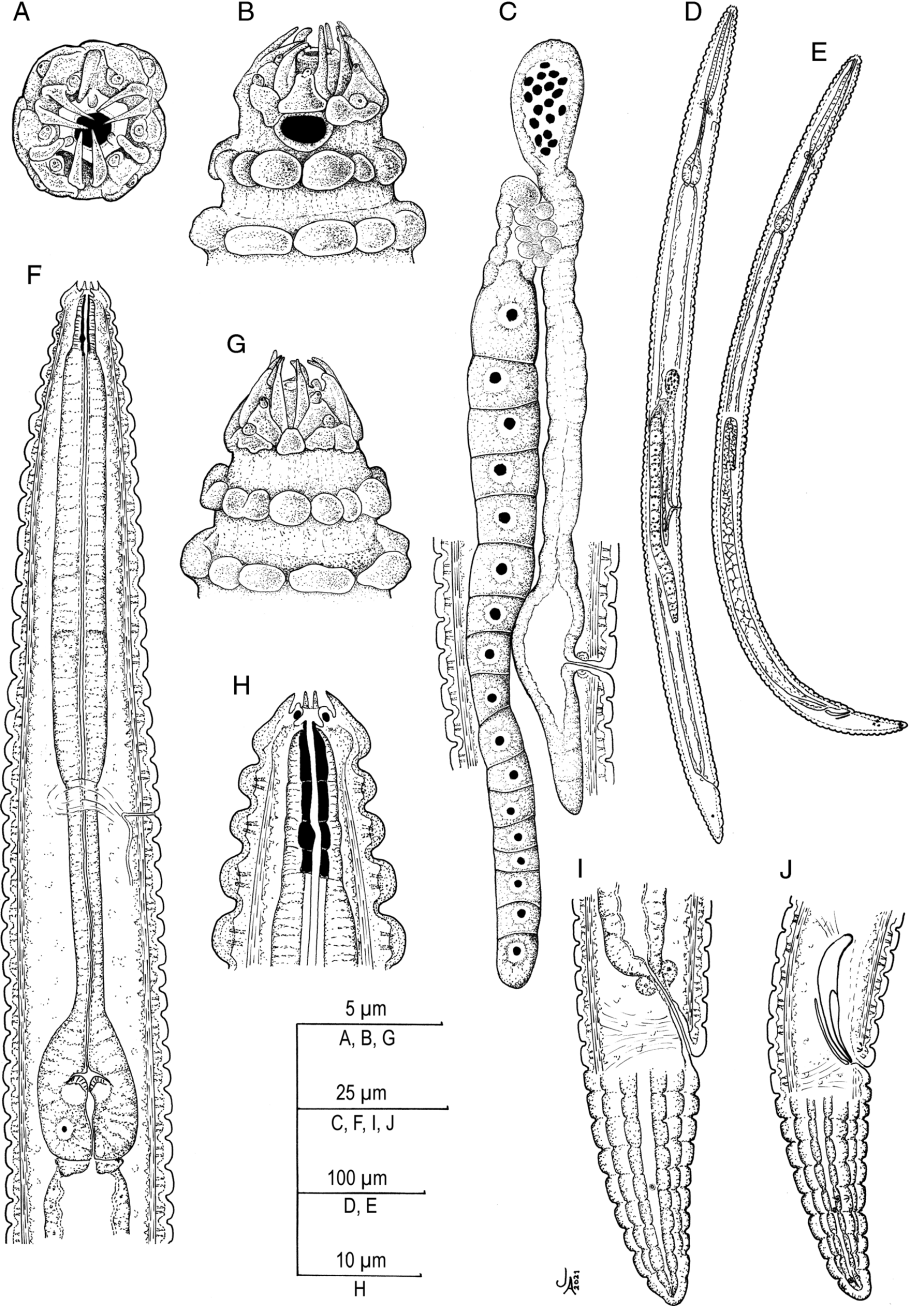
*Spinocephalus tessellatus* n. gen., n. sp. (line drawing). A, B, G: Lip region in frontal, lateral and ventral views, respectively; C: Female genital system; D: Entire female; E: Entire male; F: Neck; H: Stoma; I: Female posterior end; J: Male posterior end.

**Figure 2:**
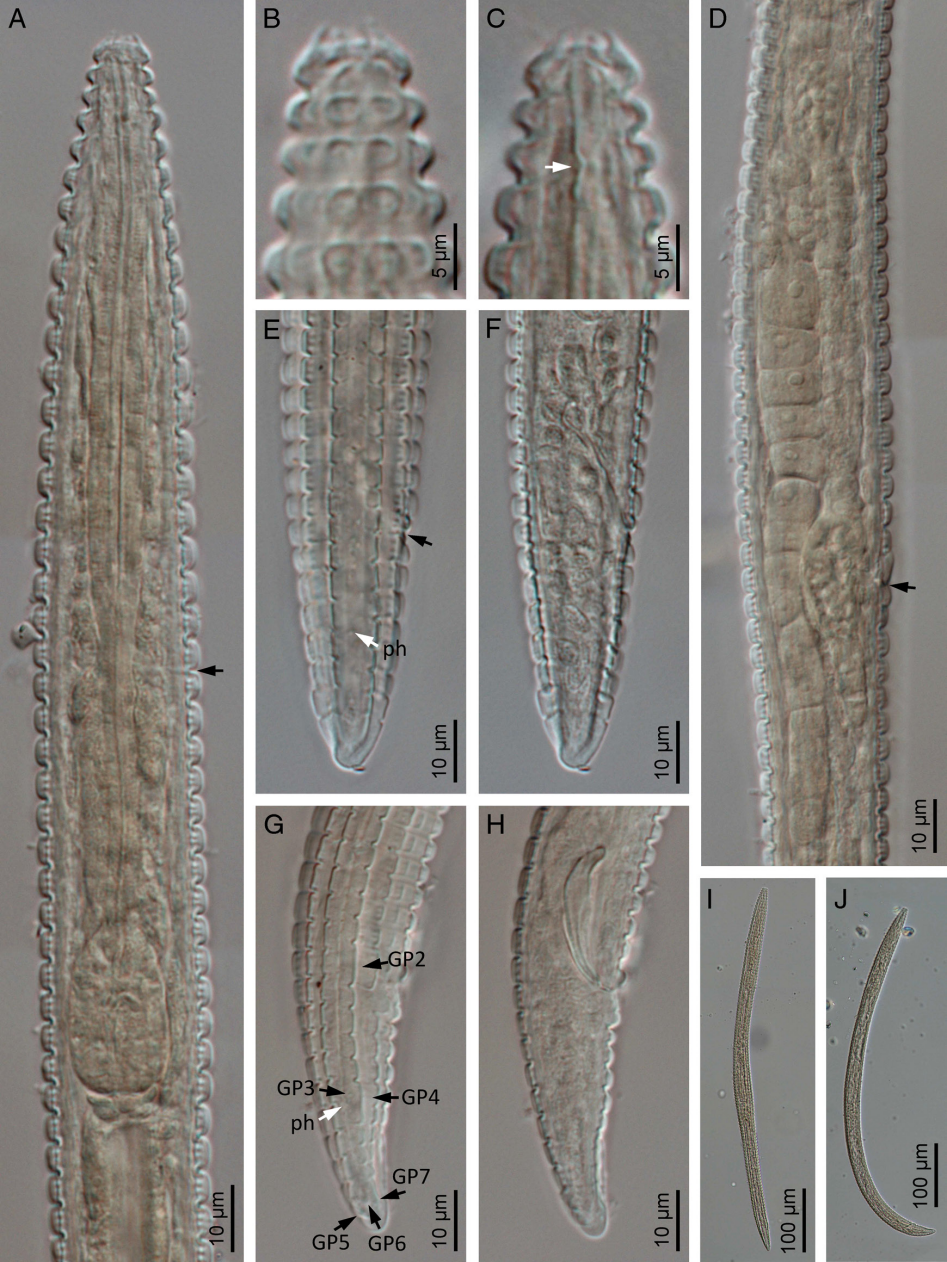
*Spinocephalus tessellatus* n. gen., n. sp. (light microscopy). A: Neck (arrow pointing the excretory pore); B, C: Anterior end at cuticle and stoma levels, respectively (arrow pointing the metastomatal dorsal tooth); D: Female genital system (arrow pointing the vulva); E, F: Female posterior end at cuticle and rectum levels, respectively (black arrow pointing the anus, white arrow pointing the phasmid, ph); G, H: Male posterior end at cuticle and spicules level (black arrows pointing the genital papillae, GP, white arrow pointing the phasmid, ph); I: Entire female; J: Entire male.

**Figure 3:**
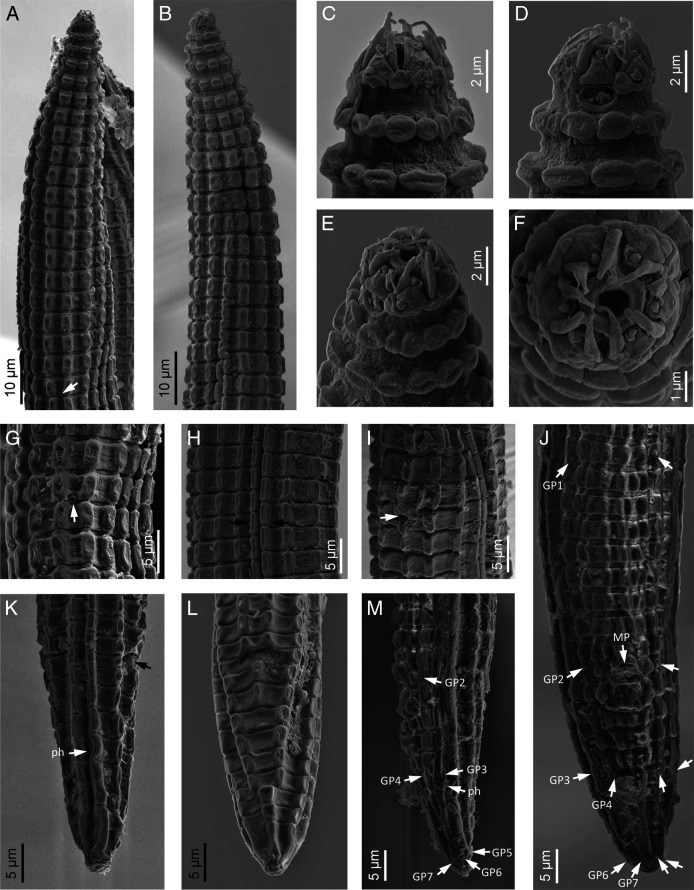
*Spinocephalus tessellatus* n. gen., n. sp. (scanning electron microscopy). A, B: Neck at ventral and lateral views, respectively (arrow pointing the excretory pore); C–F: Lip region in dorsal, lateral, subdorsal and frontal views, respectively; G: Excretory pore (arrow); H: Lateral field (between arrows); I: Vulva (arrow); J, M: Male posterior end in ventral and lateral views, respectively (arrows pointing the genital papillae, GP, middle papillae, MP, and phasmid, ph); K, L: Female posterior end in lateral and ventral views, respectively (black arrow pointing the anus, white arrow pointing the phasmid, ph).

**Figure 4:**
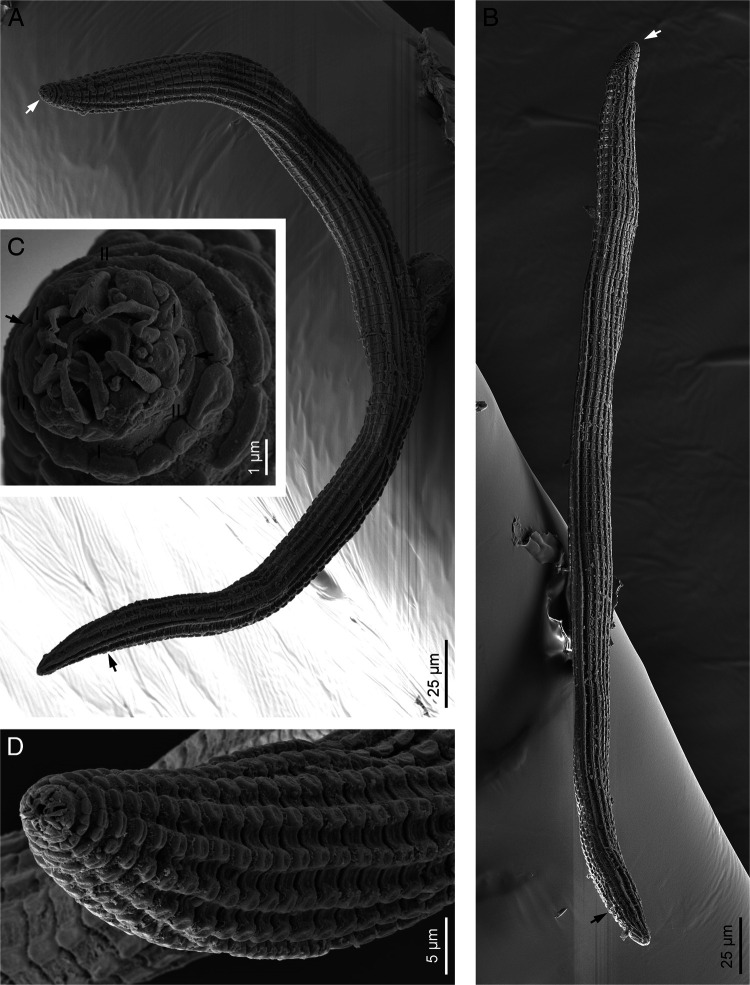
*Spinocephalus tessellatus* n. gen., n. sp. (scanning electron microscopy). A: Entire female (white arrow pointing the anterior end, black arrow pointing the anus); B: Entire male (white arrow pointing the anterior end, black arrow pointing the cloacal aperture); C: Lip region in frontal view (arrows pointing the phasmids, I =  primary axil, II = secondary axil); D: Neck region.

*Spinocephalus tessellatus* n. gen., n. sp.

(Figs. 1–4 and Table 1).

**Table 1.** Morphometrics of *Spinocephalus tessellatus* n. gen., n. sp

Sex	Holotype	Paratypes	
*n*	Female	11 females	6 males
L	575	612.4 ± 34.8 (552–668)	596.0 ± 25.3 (578–645)
a	21.3	21.0 ± 0.6 (20.0–22.0)	24.1 ± 2.5 (20.2–27.9)
b	3.7	4.3 ± 0.1 (4.0–4.5)	3.5 ± 0.2 (3.2-3.8)
c	14.0	15.4 ± 1.2 (13.8–17.7)	14.5 ± 0.6 (13.9–15.7)
c'	2.3	2.0 ± 0.1 (1.8–2.3)	2.0 ± 0.1 (2.0–2.2)
V	62	61.2 ± 0.9 (60–63)	–
Lip region width	9	5.5 ± 0.4 (5–6)	5.1 ± 0.4 (5–6)
Stoma length	13	12.1 ± 0.4 (11–13)	11.5 ± 0.5 (11–12)
Corpus length	73	70.6 ± 4.3 (63–76)	88.1 ± 7.1 (81–101)
Isthmus length	46	45.2 ± 1.3 (44–48)	45.3 ± 2.4 (42–49)
Bulbus length	25	25.9 ± 1.5 (24–28)	24.3 ± 0.6 (24–25)
Pharynx length	144	141.8 ± 4.7 (134–148)	157.8 ± 6.9 (150–168)
Neck length	157	154.0 ± 4.9 (146–161)	169.3 ± 7 (162–180)
Nerve ring-ant. end	89	98.7 ± 8.9 (85–112)	100.8 ± 9.1 (86–110)
Excretorypore-ant. end	94	104.2 ± 5.6 (94–113)	100.2 ± 4.5 (95–106)
Deirid-ant. end	117	110.0 ± 4.2 (107–113)	122.0 ± 3.6 (118–125)
Cuticle thickness	3	2.6 ± 0.1 (2–3)	2.2 ± 0.2 (2–3)
Annuli width	4	5.1 ± 0.5 (4–6)	5.0 ± 0.2 (4–5)
Body width neck base	26	27.6 ± 1.5 (26–30)	23.8 ± 1.7 (21–27)
Body width mid-body	27	29.0 ± 1.7 (26–32)	24.9 ± 2.7 (21–29)
Lateral field width	7	8.2 ± 0.8 (7–10)	7.3 ± 0.5 (7–8)
Ovary length	120	122.0 ± 20.7 (96–142)	–
Oviduct length	9	11.2 ± 2.2 (8–13)	–
Spermatheca length	32	31.6 ± 7.1 (23–39)	–
Uterus length	70	68.2 ± 16.9 (43–79)	–
Vagina length	9	7.6 ± 0.7 (7–9)	–
Post-vulval uterinesac	29	25.7 ± 1.4 (23–28)	–
Vulva-ant. end	357	375 ± 25.8 (331–418)	–
Rectum length	23	19.4 ± 1.9 (17–22)	–
Body width anus	18	18.9 ± 1.1 (17-21)	19.8 ± 0.6 (19–21)
Tail length	41	39.8 ± 2.6 (37–44)	40.9 ± 0.6 (40–42)
Phasmid-anus distance	18	22.4 ± 10.4 (17–41)	19.1 ± 2.4 (18–23)
Spicules length	–	–	24.5 ± 0.8 (24–26)
Gubernaculum length	–	–	12.4 ± 1.1 (11–14)

**Note:** Measurements in μm and in the form: mean ± s.d. (range) where available.

### Description

#### Adults

Stout to moderately slender nematodes, body length 0.55–0.67 mm. Habitus slightly ventrally curved in females and J-shaped in males after fixation. Cuticle deeply tessellated, having deep transversal and longitudinal incisures dividing the cuticle in blocks, the first annuli with wider than long blocks, later quadrangular and posteriorly longer than wide until the tail end. Lateral field with three longitudinal incisures or two narrow and tessellated wings, occupying 26–31% of mid-body diam., extending to tail end. Anterior body end narrower with lip region continuous with adjacent body, having six lips, slightly triangular, the lateral ones larger, and bearing six smaller labial and four larger cephalic sensilla; primary axils deep, V-shaped, bearing two conoid-elongate guard processes originating from each lip; secondary axils deep, U-shaped, with one thorn-like process (labial probolae?) in lateral view bearing a small rounded protuberance fused at an oral plate having triradiate symmetry developing more acute margin toward each primary axil. Amphid openings clearly visible, large, rounded to slightly oval. Oral opening hexagonal. Stoma cephaloboid with cheilostom bearing minute and rounded rhabdia, gymnostom very short, stegostom with minute discernible rhabdia being prostegostom longer and metastegostom bearing a minute dorsal tooth. Pharynx cephaloboid with pharyngeal corpus subcylindrical with metacorpus not well differentiated, isthmus unusually slender, 1.4–1.7 times corpus length, basal bulb pyriform, with well-developed valvular apparatus (grinder). Cardia conoid, surrounded by intestinal tissue. Nerve ring at 53–70% of neck length, surrounding the isthmus. Excretory pore at 58–74% of neck length, situated at level of isthmus, 18–25 annuli from anterior end. Deirids poorly discernible, at 72–75% of neck length, situated at level of isthmus, 23–29 annuli from anterior end. Intestine without distinct specialization.

#### Female

Reproductive system cephaloboid, monodelphic-prodelphic having a globular sac-like spermatheca, in dextral position to intestine; ovary short, lacking flexures, with oocytes in one row; oviduct very short, areolate; spermatheca well-developed, as width as corresponding body diam., sometimes with sperm, this with 3–4 µm long; uterus about three times as long as corresponding body diam., distally tubular with thick walls and proximally swollen with thin walls; post-vulval uterine sac poorly developed, 0.8–1.0 times body diam., proximally swollen with thin walls and distally narrower lacking lumen; vagina short, extending inward 22–28% of body diam.; vulva very reduced, oval. Rectum 0.8–1.3 times anal body diam., with three small gland-like cells distinguishable around intestine-rectum junction. Tail conoid with small rounded terminus, with 9–12 annuli at ventral side. Phasmids located at 43–47% of tail length.

#### Male

General morphology similar to female. Reproductive system monorchic, dextral in position, with testis reflexed ventral anteriorly. Spicules paired and symmetrical, with rounded, ventral bent manubrium, conoid calamus and slightly ventrally curved lamina with very small dorsal hump, poorly developed ventral wing and acute tip. Gubernaculum well developed, ventrally curved, 0.5–6.0 times the spicule length, with thin manubrium and corpus, and crura well developed. Three small gland-like cells distinguishable around beginning of cloaca. Genital papillae one pair pre-cloacal, one pair ad-cloacal and five pairs post-cloacal arranged as follows: two in middle tail region (one lateral located at lateral field level and one subventral), and three pairs near tail terminus (one subdorsal, one lateral, and one subventral). Tail conical and ventrally curved with small rounded terminus. Phasmids located at 43–55% of tail length, slightly posterior to genital papillae GP3.

### Molecular characterization

A sequence with 6,258 bp (MZ621174) of the 18S rDNA and two sequences lacking differences with 936 bp (MZ621172, MZ621173) of the 28S rDNA fragment were obtained for *Spinocephalus tessellatus* n. gen., n. sp. This genus and species show a higher similarity with some species of the genera *Acromoldavicus* and *Nothacrobeles* Allen and Noffsinger, 1971 maintain a common aligned fragment with 657 bp. With respect to *Nothacrobeles abolafiai* Mehdizadeh and Shokoohi, 2019 (KC182515) the common 28S fragments show 52 bp (7.9%) differences (substitutions, deletions, or insertions), 72 bp (11.1%) differences with *N. cancellatus* (Thorne, 1925) Ruiz-Cuenca and Abolafia, 2020 (HM439765) and 137 bp (20.8%) differences with *N. hebetocaudatus* Abolafia, Divsalar, Panahi and Shokoohi, 2014 (KJ508411). With respect to *Acromoldavicus mojavicus* (AY027536, DQ145626) shows 66 bp (10.0%) differences, while with *A. skrjabini* (AY027535) shows 75 bp (11.4%) differences.

### Type locality and habitat

The specimens were collected at sandy soil in the rhizosphere of *Tamarix passerinoides* Delile ex Desv. in Shush (ancient Persian city of Susa, GPS coordinates: 32°17.28′N, 48°25.07′E), Khuzestan province, Iran.

### Type material

Six females (holotype and paratypes) and five males (paratypes) deposited in the nematode collection of the Departamento de Biología Animal, Biología Vegetal y Ecología, Universidad de Jaén, Spain. One female and one male (paratypes) deposited in the nematode collection of the Department of Plant Protection, College of Agriculture, University of Zanjan, Zanjan, Iran.

### Differential diagnosis

The body length of the new species range from 0.55 –0.67 mm long in females and 0.58–0.65 mm long in males. The cuticle shows deep transversal and longitudinal incisures which divides the cuticle in blocks, or tessellated, and the lateral fields with two tessellated longitudinal wings or three longitudinal incisures. The lip region has six triangular lips appearing the primary axils deeper with V-shaped bearing two conoid-elongate guard processes originating from each lip while the secondary axils are deeper with U-shaped bearing one thorn-like process (labial probolae?) observed in lateral view and bearing a small rounded protuberance fused at an oral plate having triradiate symmetry which develops a more acute margin toward each primary axil. The oral opening is hexagonal The amphids are large and rounded to slightly oval. The stoma is cephaloboid with cheilostom bearing minute and rounded rhabdia. The pharynx is cephaloboid with subcylindrical corpus and very long isthmus being 1.4–1.7 times corpus length. The nerve ring surrounds the isthmus and the excretory pore appears at 65–73% of neck length at isthmus level. The female reproductive system is monodelphic-prodelphic with spermatheca as long as the body diam. and post-vulval uterine sac 0.8–1.0 times body diameter. The female tail is conoid with small rounded terminus. The male reproductive system is monorchic having spicules 24–26 µm long and gubernaculum 11–14 µm long. The male tail is conical and ventrally curved ending in a small, rounded terminus.

### Etymology

The specific name refers to the presence of cuticular blocks or tessellation (Latin *tessella* = mosaic pavers). Zoobank code urn:lsid:zoobank.org:pub:53D4EE5A-A6B4-4A91-9896-88A5A6134D6C

### Relationships

The morphological analysis of *Spinocephalus tessellatus* n. gen., n. sp. shows the presence of an unusual lip pattern which structure is not similar to other genera belonging to the superfamily Cephaloboidea. The presence of a stoma with small rhabdia and sac-like spermatheca clearly indicates its relationship with other species of the infraorder Cephalobomorpha. However, the absence of clear labial probolae and the unusual structure of the lips make difficult to know its position at family level.

**Figure 5:**
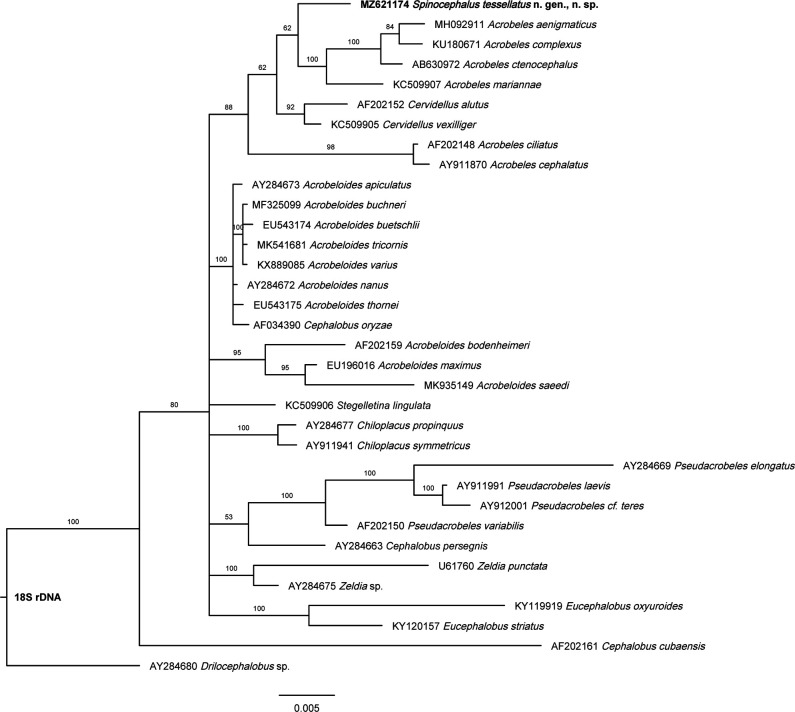
Bayesian inference tree showing the phylogenetic position of *Spinocephalus tessellatus* n. gen., n. sp. and its related taxa based on sequences of the 18S rDNA region. Bayesian posterior probabilities (%) are given for each clade. Scale bar shows the number of substitutions per site.

**Figure 6:**
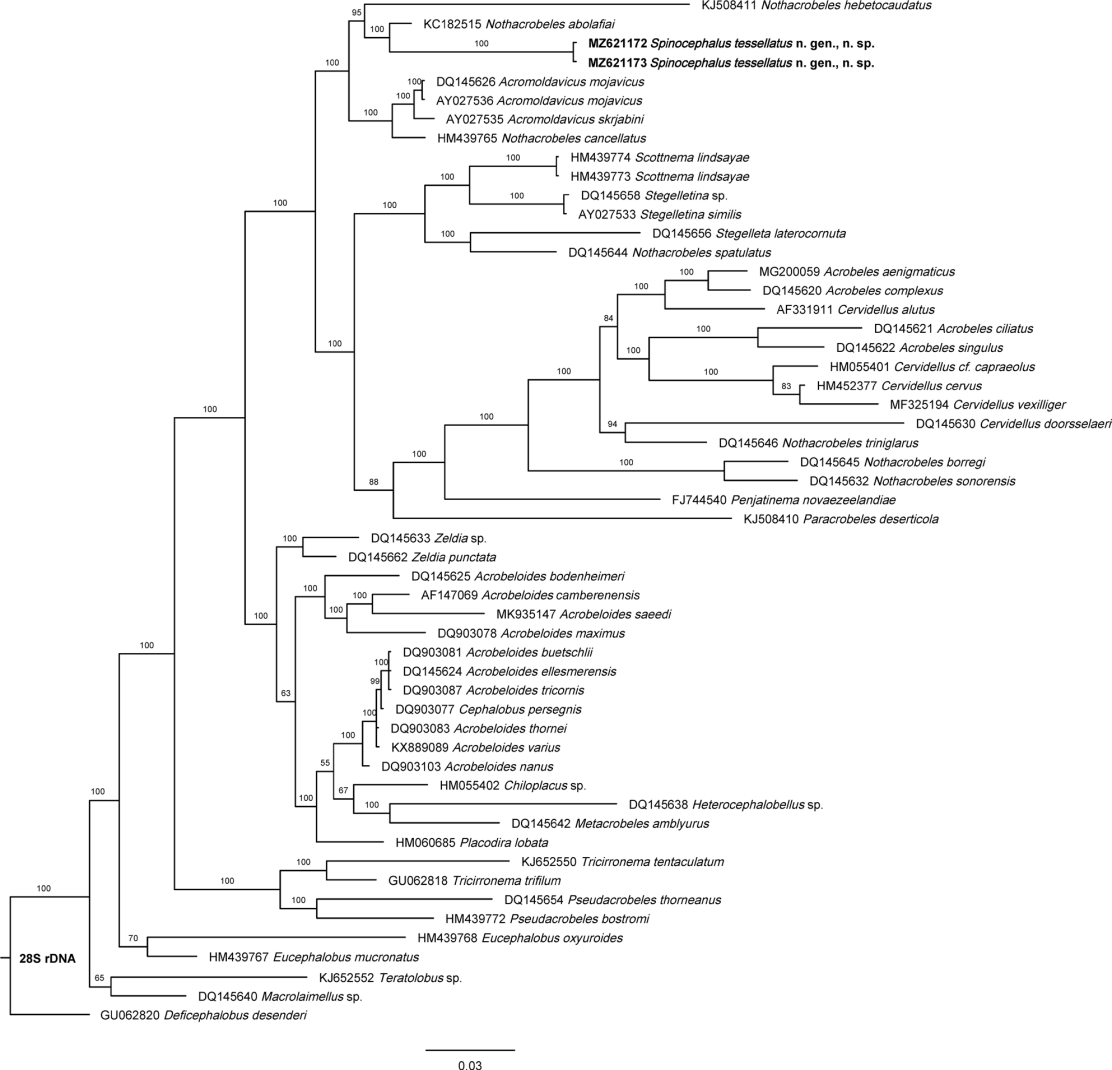
Bayesian inference tree showing the phylogenetic position of *Spinocephalus tessellatus* n. gen., n. sp. and its related taxa based on sequences of the 28S rDNA region. Bayesian posterior probabilities (%) are given for each clade. Scale bar shows the number of substitutions per site.

**Figure 7:**
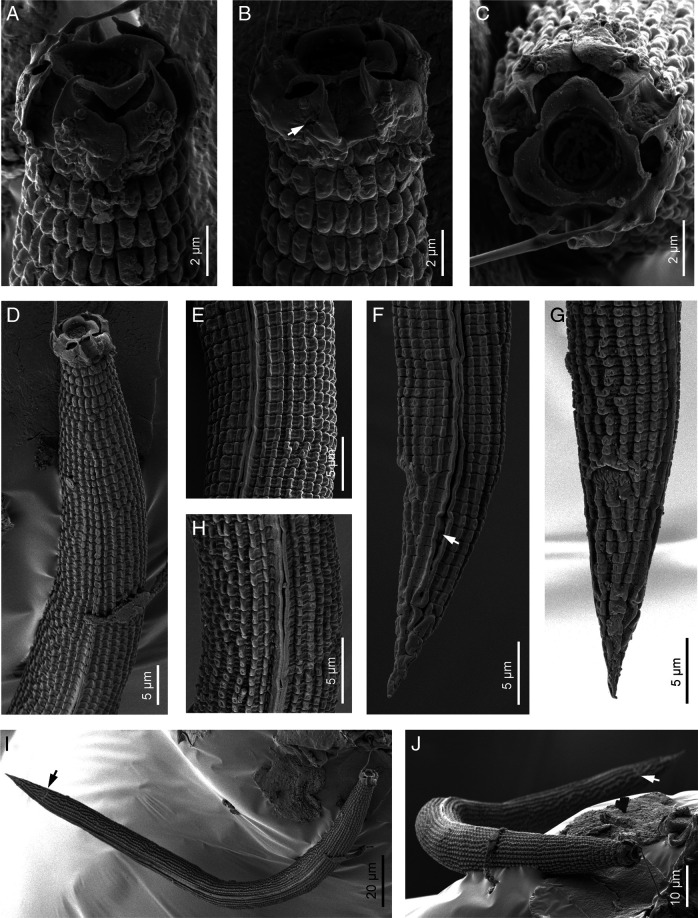
*Acromoldavicus skrjabini* (Nesterov and Lisetskaya, 1965) Nesterov, 1970 from Santa Catalina Mountain, Jaén, Spain (scanning electron microscopy, juvenile). A–C: Lip region in dorsal, left lateral and frontal views, respectively; D: Neck region; E, H: Lateral field; F, G: Posterior end in lateral and ventral views, respectively (arrow pointing the phasmid); I, J: Entire body (arrow pointing the anus).

**Figure 8:**
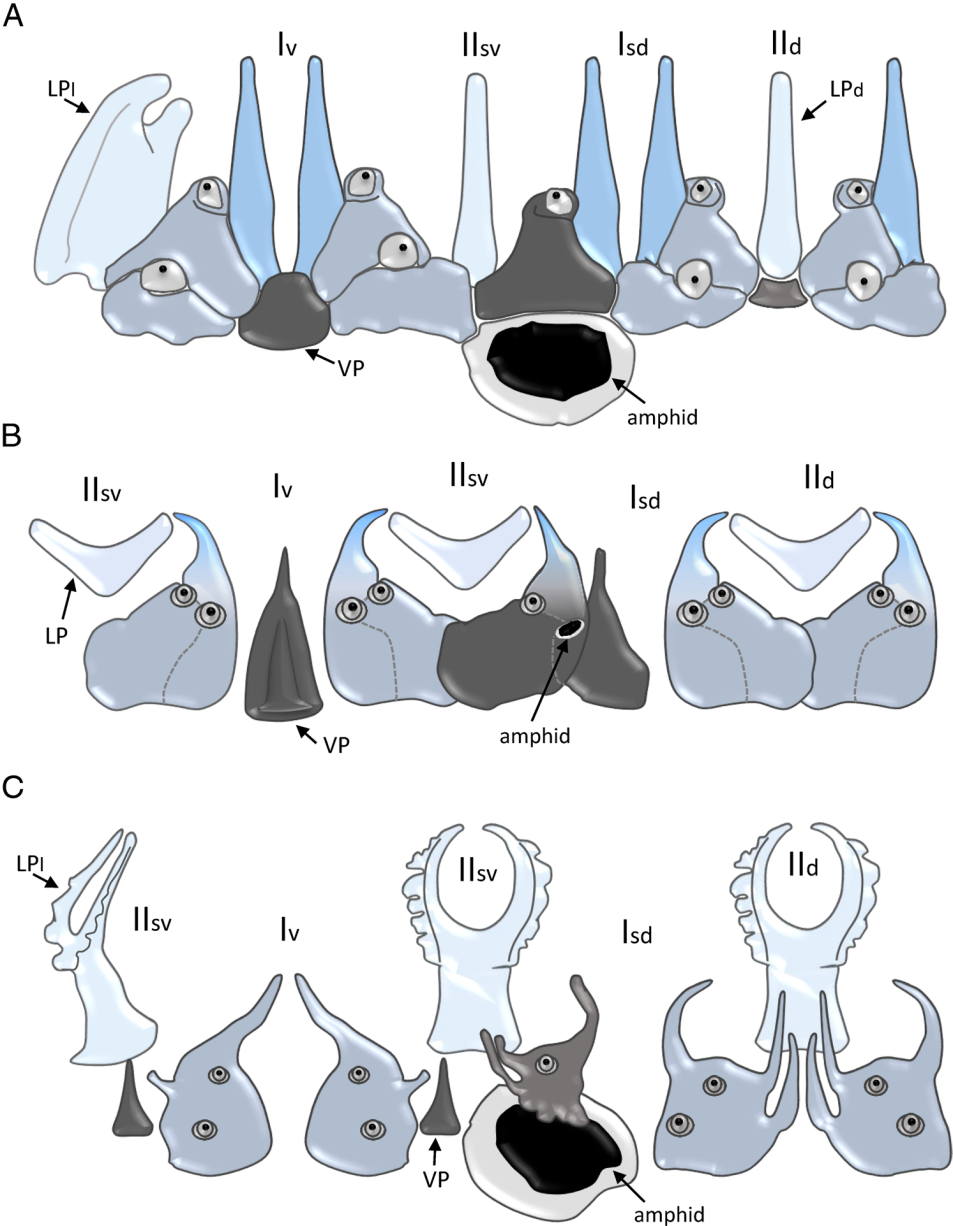
Schematic pattern of the labial region of *Spinocephalus tessellatus* n. gen., n. sp. (A), *Acromoldavicus skrjabini* (Nesterov and Lisetskaya, 1965) Nesterov, 1970 (B) and *Chilodellus eremus* Boström and Holovachov, 2012 (C). Isd: subdorsal primary axil; Iv: ventral primary axil; IId: dorsal secondary axil; IIsv: subventral secondary axil; LP: labial probola; LPd: labial probolae in dorsal view; LPl: labial probolae in lateral view; VP: ventral process.

Molecular analysis based on 18S rDNA (Fig. 5) does not resolve well the phylogenetic relationships of *Spinocephalus* n. gen. because few genera of the superfamily Cephaloboidea are sequenced currently, appearing the new genus grouped to some species of the genus *Acrobeles* von Linstow, 1877 although they do not maintain closemorphological similarities. On the other hand, the 28S rDNA phylogenetic tree (Fig. 6) shows that the new genus is related with species of the genera *Nothacrobeles* and *Acromoldavicus*, both containing species with tessellated cuticle such as *N. cancellatus* (see Karegar et al., 1997, 1998) and *Acromoldavicus* species (Fig. 7). With respect to the lip region, the lip pattern of *Spinocephalus* n. gen. (Fig. 8A) resembles slightly to that in *Acromoldavicus* (Fig. 8B). Thus, the ventral triangular process of *Acromoldavicus* could be homologous to the polygonal plate appearing in *Spinocephalus* n. gen., while the elongate process visible at the primary axils could be homologous to the acute tip of each lip present in *Acromoldavicus*. However, the lip pattern observed in the *Nothacrobeles* species, with dentate lips, is very different.

Other species with an irregular lip pattern is *Chilodellus eremus* Boström and Holovachov, 2012 (Fig. 8C). This species has very large amphid openings, similar to *Spinocephalus tessellatus* n. gen., n. sp., and lips with long, acute processes, the lateral ones more reduced, which have a large amphid opening. However, the labial probolae have very different morphology with bifurcate distal part with pinnate outer margin.

According to this, *Spinocephalus tessellatus* n. gen., n. sp. is tentatively located in the family Cephalobidae, subfamily Cephalobinae instead of the family Elaphonematidae, subfamily Kirjanoviinae.
